# Supplementary material for: MiR-146a wild-type 3′ sequence identity is dispensable for proper innate immune function in vivo
Source: Life Sci Alliance. 2019 Feb 18;2(1):e201800249. doi: 10.26508/lsa.201800249 (PMC6379685; doi:10.26508/lsa.201800249)
Supplement: Supplementary file 4 [file LSA-2018-00249_TableS4.docx]

**Antibody and Oligonucleotide Table**

| **Antibodies** | | |
| --- | --- | --- |
| **Antibody** | **Supplier** | **Catalog Number** |
| FITC hamster monoclonal anti-TCRβ | BD Pharmingen | Cat# 553171 |
| PerCP-Cy™5.5 rat monoclonal anti-CD4 | BD Pharmingen | Cat# 561115 |
| PE-Cy™5 rat monoclonal anti-CD8α | BD Pharmingen | Cat# 553034 |
| APC rat monoclonal anti-CD62L | BD Pharmingen | Cat# 561919 |
| PE rat monoclonal anti-CD44 | BD Pharmingen | Cat# 553134 |
| PE-Cy™7 rat monoclonal anti-CD69 | BD Pharmingen | Cat# 552879 |
| PerCP-Cy™5.5 rat monoclonal anti-F4/80 | Thermo Fisher Scientific | Cat# 45-4801-80 |
| APC rat monoclonal anti-Ly6G and Ly6C | BD Pharmingen | Cat# 561083 |
| FITC rat monoclonal anti-CD11b | BD Pharmingen | Cat# 557396 |
| APC hamster monoclonal anti-CD3ε | BD Pharmingen | Cat# 553066 |
| PE rat monoclonal anti-B220 | BD Pharmingen | Cat# 553089 |
| PE hamster monoclonal anti-CD11c | BioLegend | Cat# 117318 |
| Rabbit anti-mouse IRAK1 mAb | Cell Signaling Technology | Cat# 4504S |
| Hamster anti-mouse TRAF6 mAb | MBL | Cat# M092-3 |
| Mouse anti-mouse GAPDH mAb | EMD Millipore | Cat# MAB374 |
| Donkey anti-rabbit IgG-HRP | Sigma-Aldrich | Cat# NA9340V-1ML |
| Goat ant-Armenian hamster IgG-HRP | Santa Cruz Biotechnology | Cat# sc-2443 |
| Sheep anti-mouse IgG-HRP | Genesee Scientific | Cat# 84-848 |

| **Oligonucleotides** | | |
| --- | --- | --- |
| **Name** | **Supplier** | **Sequence** |
| Arm 1 Fwd Primer | Sigma-Aldrich | 5ʹ*-*ATCGTAAAGCTTCCCA GGTACTGGGAAGAACA*-*3ʹ |
| Arm 1 Rev primer | Sigma-Aldrich | 5ʹ-ATCGTAGCGGCCGCCA CCTCAGCAGACCATGCTA-3ʹ |
| Arm 2 Fwd primer | Sigma-Aldrich | 5ʹ-ATCGTAGGATCCGAGA  GACACAGGATTGCCAAGCAGTGATTTC-3ʹ |
| Arm 2 Rev primer | Sigma-Aldrich | 5ʹ-ATCGTAGCGGCCGCCA CTGGCTAAGGGTCGGATA-3ʹ |
| WT Northern probe | Sigma-Aldrich | 5ʹ-CGCATTATTACTCACG  GTACGA -3ʹ |
| 3ʹF Northern probe | Sigma-Aldrich | 5ʹ-AAGGACACTTATTCAG  TTCTCA -3ʹ |
| Arm 1 (Internal) Southern probe PCR FWD | Sigma-Aldrich | 5ʹ-GCCCTCTATGGGGTCT  TCTC-3ʹ |
| Arm 1 (Internal) Southern probe PCR REV | Sigma-Aldrich | 5ʹ-TCTGGTCCTTTGT-3ʹ |
| Arm 2 (External) Southern probe PCR FWD | Sigma-Aldrich | 5ʹ-CAGGAACTCCTCTCCA  ACCA-3ʹ |
| Arm 2 (External) Southern probe PCR REV | Sigma-Aldrich | 5ʹ-CACTGGCTAAGGGTCG  GATAT CCTGCT-3ʹ |
| Genotyping FWD primer | Sigma-Aldrich | 5ʹ-GGAAATCACTGCTTGG  CAAT-3ʹ |
| Genotyping REV primer | Sigma-Aldrich | 5ʹ-CTGAGTGGTTCTTGCT  GCTG-3ʹ |
| miScript qRT-PCR WT primer | Qiagen | Proprietary |
| miScript qRT-PCR 3ʹF primer | Qiagen | Proprietary |
| *mmu-mir-146a-5p*^WT^ mimic | Sigma-Aldrich | 5ʹ-pUGAGAACUGAAUUC  CAUGGG [dT][dT]-3ʹ |
| *mmu-mir-146a-3p*^WT^ mimic | Sigma-Aldrich | 5ʹ-pCCCAUGGAAUUCAG  UUCUCA [dT][dT]-3ʹ |
| *mmu-mir-146a-5p^3ʹF^* mimic | Sigma-Aldrich | 5ʹ-pUGAGAACUGAAUAA  GUGUCC [dT][dT]-3ʹ |
| *mmu-mir-146a-3p^3ʹF^* mimic | Sigma-Aldrich | 5ʹ-pGGACACUUAUUCAG  UUCUCA [dT][dT]-3ʹ |
| anti-CXCR4 siRNA sense strand | Sigma-Aldrich | 5ʹ-pGUUUUCACUCCAGC  UAACA [dT][dT]-3ʹ |
| anti-CXCR4 siRNA antisense strand | Sigma-Aldrich | 5ʹ-pUGUUAGCUGGAGUG  AAAAC [dT][dT]-3ʹ |
| *Irak1* 3ʹ UTR FWD primer | Sigma-Aldrich | 5ʹ-ACTGACCTCGAGTTCA  CTCTGA CAAATCCCTCA-3ʹ |
| *Irak1* 3ʹ UTR REV primer | Sigma-Aldrich | 5ʹ-ACTGACGCGGCCGCG  AGCAATCGGGTGCTCATAC-3ʹ |
| *Traf6* 3ʹ UTR FWD primer | Sigma-Aldrich | 5ʹ-ACTGACCTCGAGTTTT  GTCCGTGTACTTTACTGT-3ʹ |
| *Traf6* 3ʹ UTR REV primer | Sigma-Aldrich | 5ʹ-ACTGACGCGGCCGCCA  CTGCTCGGGTCTCTGATT-3ʹ |
| *Traf6* QuikChange 5ʹ primer #1 | Sigma-Aldrich | 5ʹ-GAAATTCACAGGAGTC  AACTAAATGTCTTCTCAACACCAGAGCGGTAACTTCTC-3ʹ |
| *Traf6* QuikChange 3ʹ primer #1 | Sigma-Aldrich | 5ʹ-GAGAAGTTACCGCTCT  GGTGTTGAGAAGACATTTAGTTGACTCCTGTGAATTTC-3ʹ |
| *Traf6* QuikChange 5ʹ primer #2 | Sigma-Aldrich | 5ʹ-AACTATAGAGGAGGG  AGTACTTGTCTTCTTAAGCCTTCCAGGGATCATGG-3ʹ |
| *Traf6* QuikChange 3ʹ primer #2 | Sigma-Aldrich | 5ʹ-CCATGATCCCTGGAA  GGCTTAAGAAGACAAGTACTCCCTCCTCTATAGTT-3ʹ |
| *Traf6* QuikChange 5ʹ primer #3 | Sigma-Aldrich | 5ʹ-CTAACTAGTCGTAGG  GCTCCCTGTCTTCTAGACTCCAAAGTACTGTATAT-3ʹ |
| *Traf6* QuikChange 3ʹ primer #3 | Sigma-Aldrich | 5ʹ-ATATACAGTACTTTGG  AGTCTAGAAGACAGGGAGCCCTACGACTAGTTAG-3ʹ |
| *Nop14* FWD primer | Sigma-Aldrich | 5ʹ-ATCGATCGCTCGAGGC  CAGATGTTTCCTGAGTCTGC-3ʹ |
| *Nop14* REV primer | Sigma-Aldrich | 5ʹ-ATTATACAGGGCCCTC  CAACCCTGGAAACGCTGC-3ʹ |
| *Terf1* 3ʹ UTR FWD primer | Sigma-Aldrich | 5ʹ-ATCGATCGCTCGAGGA  GGCTGGATGGGTTTGATTC-3ʹ |
| *Terf1* 3ʹ UTR REV primer | Sigma-Aldrich | 5ʹ-ATCGATCGGGGCCCGG  CAGCAAATTCAAGAGAACAG-3ʹ |
| *Dhrs13* 3ʹ UTR FWD primer | Sigma-Aldrich | 5ʹ-ATCGATCGCTCGAGCT  TTCCTAACCCCCGGGATAG-3ʹ |
| *Dhrs13* 3ʹ UTR REV primer | Sigma-Aldrich | 5ʹ-ATCGATCGGGGCCCCA  CAGTCAGCTCGCTTTATTCC-3ʹ |
| *Gemin6* 3ʹ UTR FWD primer | Sigma-Aldrich | 5ʹ-ATCGATCGCTCGAGGA  GGCCAAGCACTGTGAACATG-3ʹ |
| *Gemin6* 3ʹ UTR REV primer | Sigma-Aldrich | 5ʹ-ATCGATCGGGGCCCAG  TCAAAAGAACCCGAAACGG-3ʹ |
| *Mphosph6* 3ʹ UTR FWD primer | Sigma-Aldrich | 5ʹ-ATCGATCGCTCGAGAA  TGGATGCCTCCGATACAGG-3ʹ |
| *Mphosph6* 3ʹ UTR REV primer | Sigma-Aldrich | 5ʹ-ATCTATCAGGGCCCCA  CAAACCCCACACTACTCAAC-3ʹ |
| *Has1* 3ʹ UTR FWD primer | Sigma-Aldrich | 5ʹ-ATCGATCGCTCGAGGT  CTGGACATGAAGATGCAGC-3ʹ |
| *Has1* 3ʹ UTR REV primer | Sigma-Aldrich | 5ʹ-ATCAATCAGGGCCCGC  AACAGGGAGAAAATGGAGAC-3ʹ |
| *Krt14* 3ʹ UTR FWD primer | Sigma-Aldrich | 5ʹ-ATCGATCGCTCGAGAG  CTGCTACATGCTGCTCAG-3ʹ |
| *Krt14* 3ʹ UTR REV primer | Sigma-Aldrich | 5ʹ-ATCGATCGGGGCCCGT  GCAACTCAGAAAAAGAAGC-3ʹ |
| *Psmg4* 3ʹ UTR FWD primer | Sigma-Aldrich | 5ʹ-ATCGATCGCTCGAGGC  AGCAGAAGGAAGAGTTTG-3ʹ |
| *Psmg4* 3ʹ UTR REV primer | Sigma-Aldrich | 5ʹ-ATCGATCGGGGCCCGA  GGACAAAAAACAACCTG-3ʹ |
| *Txndc9* 3ʹ UTR FWD primer | Sigma-Aldrich | 5ʹ-ATCGATCGCTCGAGGT  ACATGCTCCCGTTCTCTG-3ʹ |
| *Txndc9* 3ʹ UTR REV primer | Sigma-Aldrich | 5ʹ-ATTGATTGGGGCCCTC  CATAGGGTGTCACATCCC-3ʹ |
| *Cdk12* 3ʹ UTR FWD primer | Sigma-Aldrich | 5ʹ-ATCGATCGCTCGAGCA  CAGAGACATCAAATGCCC-3ʹ |
| *Cdk12* 3ʹ UTR REV primer | Sigma-Aldrich | 5ʹ-ATCGGCGGCCGCTGTA  CATAGACAAGGTACACCC-3ʹ |
| *Paip2* 3ʹ UTR FWD primer | Sigma-Aldrich | 5ʹ-ATCGATCGCTCGAGGG  CCGCTTTTGGTGGATGTAG-3ʹ |
| *Paip2* 3ʹ UTR REV primer | Sigma-Aldrich | 5ʹ-ATCGATCGGGGCCCCA  CACTGAACACGTATGGC-3ʹ |
| *Cdk5* 3ʹ UTR FWD primer | Sigma-Aldrich | 5ʹ-ATCGATCGCTCGAGTT  TAAGCCTCCCTCCCAGGAG-3ʹ |
| *Cdk5* 3ʹ UTR REV primer | Sigma-Aldrich | 5ʹ-ATTGATTGCGGCCGCT  TGAACTCACTCCCAAGCCC-3ʹ |
| *Ceacam1* 3ʹ UTR FWD primer | Sigma-Aldrich | 5ʹ-ATCGATCGCTCGAGGC  ATAATCTGTCCGTCTGTC-3ʹ |
| *Ceacam1* 3ʹ UTR REV primer | Sigma-Aldrich | 5ʹ-ATTGATTTGGGCCCAA  ATAATCCAGCATTGCGTGGG-3ʹ |
| *Trmt44* 3ʹ UTR FWD primer | Sigma-Aldrich | 5ʹ-ACCGCTCGCTCGAGGT  CACCTCTTCAGTGTTCTC-3ʹ |
| *Trmt44* 3ʹ UTR REV primer | Sigma-Aldrich | 5ʹ-ATTTATGCGGCCGCGA  AACCACCACCCAAAGCAG-3ʹ |
| *Gramd3* 3ʹ UTR FWD primer | Sigma-Aldrich | 5ʹ-ACCGGCCGCTCGAGGC  TGGGACCAACATAAATAC-3ʹ |
| *Gramd3* 3ʹ UTR REV primer | Sigma-Aldrich | 5ʹ-ATTTAGCGGCCGCGCT  AGGGTGCCTTAGGCAATG-3ʹ |
| *Mblac1* 3ʹ UTR FWD primer | Sigma-Aldrich | 5ʹ-ATCGATCGCTCGAGCT  GGACTCCAGTCAGGAAAG-3ʹ |
| *Mblac1* 3ʹ UTR REV primer | Sigma-Aldrich | 5ʹ-ATTGATTTGGGCCCAA  ATCAGGATGGCTCAGCAG-3ʹ |
